# Prognostic correlation analysis of colorectal cancer patients based on monocyte to lymphocyte ratio and folate receptor-positive circulating tumor cells and construction of a machine learning survival prediction models

**DOI:** 10.3389/fonc.2025.1531836

**Published:** 2025-05-01

**Authors:** Siying Pan, Chi Lu, Hongda Lu, Hongfeng Zhang

**Affiliations:** ^1^ Department of Oncology, The Central Hospital of Wuhan, Tongji Medical College, Huazhong University of Science and Technology, Wuhan, China; ^2^ Hubei Provincial Engineering Research Center of Intestinal Microecological Diagnostics, Therapeutics, and Clinical Translation, Wuhan, China; ^3^ Department of Pathology, The Central Hospital of Wuhan, Tongji Medical College, Huazhong University of Science and Technology, Wuhan, China

**Keywords:** colorectal cancer, monocyte to lymphocyte ratio (MLR), folate receptor-positive circulating tumor cells (FR+CTCs), prognostic, machine learning (ML), prediction model

## Abstract

**Purpose:**

To evaluate the prognostic value of the monocyte to lymphocyte ratio (MLR) and folate receptor-positive circulating tumor cells (FR+CTCs) in patients with colorectal cancer (CRC) and to develop predictive model for post-treatment survival using machine learning (ML) algorithms.

**Methods:**

We retrospectively analyzed 67 CRC patients treated with radical surgery or chemoradiotherapy at The Central Hospital of Wuhan from January 2020 to December 2022. MLR, neutrophil to lymphocyte ratio (NLR), platelet to lymphocyte ratio (PLR) and FR+CTCs were categorized into high and low groups and clinicopathologic features were compared. Progression-Free Survival (PFS) and Overall Survival (OS) were analyzed using COX analysis and the Kaplan-Meier survival curve. Three ML algorithms, namely, random forest (RF), support vector machine (SVM), and logistic regression (LR), were utilized to construct the predictive models, and their performance metrics including accuracy, sensitivity, specificity, positive predictive value (PPV), negative predictive value (NPV), precision, recall, F1 value, AUC, and calibration curve were compared.

**Results:**

MLR, FR+ CTCs, and T stage independently predicted PFS (P<0.05), both higher MLR and FR+CTCs levels indicating a significantly shorter PFS (P=0.004). The T stage was the only factor predictive of OS (P=0.043). NLR and PLR did not show significant prognostic effects on PFS or OS (P > 0.05). The RF model demonstrated superior performance with an accuracy of 0.63, sensitivity of 0.69, PPV of 0.75, a precision of 0.43, a recall of 0.5, and an F1 value of 0.43, outperforming the other models.

**Conclusion:**

High MLR and high FR+CTCs are associated with a poorer PFS in CRC patients, suggesting their utility in prognostic assessment. NLR and PLR did not show significant prognostic value in this study. The RF algorithm-based model showed the best predictive performance for post-radical treatment outcomes in CRC.

## 1 Introduction

Colorectal cancer (CRC) is currently one of the most common malignancies, with its incidence and mortality rates holding the third and second positions, respectively, according to the 2022 World Health Organization cancer statistics (1). Throughout the past decades, there has been a remarkable evolution in the diagnostic and therapeutic approaches to CRC, transitioning from a reliance solely on surgical intervention to a more multifaceted treatment strategy that has significantly improved the prognosis of patients. For patients who have undergone radical surgery or chemoradiotherapy, postoperative surveillance for recurrence and metastasis primarily relies on imaging techniques. However, the detection of micro-metastases through imaging remains a challenge. Therefore, there is an urgent requirement for a sensitive and simple biomarker to assist in forecasting the prognosis of CRC patients, which would be of immense value in improving survival rates.

In recent years, an increasing body of research has underscored the intimate connection between tumorigenesis/progression and systemic inflammatory response (2–4).Within the tumor microenvironment (TME), inflammation is pivotal, encompassing a mix of tumor cells, mesenchymal stromal cells, and immune cells. Various inflammatory indicators, including counts of leukocytes, lymphocytes, neutrophils, monocytes, platelets, and levels of C-reactive protein (CRP), serve as indicators of the body’s immune function and inflammatory status. Many scholars have delved into the analysis of these indicators, examining their absolute values or ratios, such as NLR (5–8), monocyte to lymphocyte ratio (MLR) (9), PLR (10), to understand their correlation with tumor prognosis. Numerous studies have demonstrated the value of NLR and PLR in CRC prognostic stratification. A key inflammatory biomarker, NLR can forecast the prognosis of patients with a variety of cancers, including breast cancer, lung cancer, hepatocellular carcinoma, and gastric cancer (5–8). PLR had been shown to be a good predictor of chemotherapy sensitivity and prognosis in patients with gastric cancer, with high PLR associated with poorer prognosis and chemotherapy resistance (10). A recent meta-analysis showed that LMR was better at predicting OS in CRC patients (AUC:0.65-0.78) than NLR (AUC: 0.60-0.75) and PLR (AUC: 0.60-0.72), but its use in combination with CTCs has not been explored (11).

Circulating tumor cells (CTCs) are characterized as those cancer cells that break away from the primary or metastatic tumor site, infiltrate the bloodstream or bone marrow, and circulate or cluster within these environments. These disseminated cancer cells can evade the immune system to form micro- metastatic clumps, which then enter the circulation and eventually establish new metastatic foci in distant tissues (12). Extensive research has been conducted on the mechanisms and underlying CTCs and their detection methods, with numerous studies indicating that CTCs are closely associated with tumor progression and can serve as a prognostic indicator for various cancers, including breast cancer, lung cancer, and CRC, among others (9, 13, 14). Folate receptors (FR), as a type of transmembrane single-chain glycoprotein with tissue specific expression, are typically not detected in the circulating cells of healthy individuals (15). In contrast, folate receptors demonstrate a high level of specificity in a variety of malignancies, such as urological tumors, pancreatic cancer, and lung cancer (16–18). The objective of this study is to assess the prognostic value of combining the MLR with the detection of FR+CTCs in patients of CRC.

Machine Learning (ML) is an important part of Artificial Intelligence (AI), with its conceptual roots dating back to the 1950s, as introduced by Arthur Samuel. ML integrates the core principles of computer science and statistics, offering a technology that excels at dissecting sample data, extracting critical factors, uncovering underlying patterns, and making predictions. The core of ML algorithms is to refine the precision of model predictions by continuously assimilating known information, honing the capability to predict novel outcomes, and perpetually refining their parameter to boost predictive accuracy (19, 20). Among the myriad of ML algorithms available, Random Forest (RF), Support Vector Machine (SVM), Logistic Regression (LR), and Decision Tree (DT) stand out for their widespread application. This study is poised to harness the power of ML algorithms to construct a prognostic model tailored for CRC patients. The model will integrate an expanding array of potent predictors to conduct an in-depth analysis of the correlations between these characteristics and patient survival outcomes. This study aims to address two key questions: (1) Compare the independent prognostic efficacy of MLR, NLR and PLR after radical treatment for CRC; (2) Explore the synergies between MLR and FR+CTCs, and develop a multi-indicator fusion machine learning model to overcome the limitations of a single inflammatory marker.

## 2 Materials and methods

### 2.1 Design and patient selection

**Figure 1:**
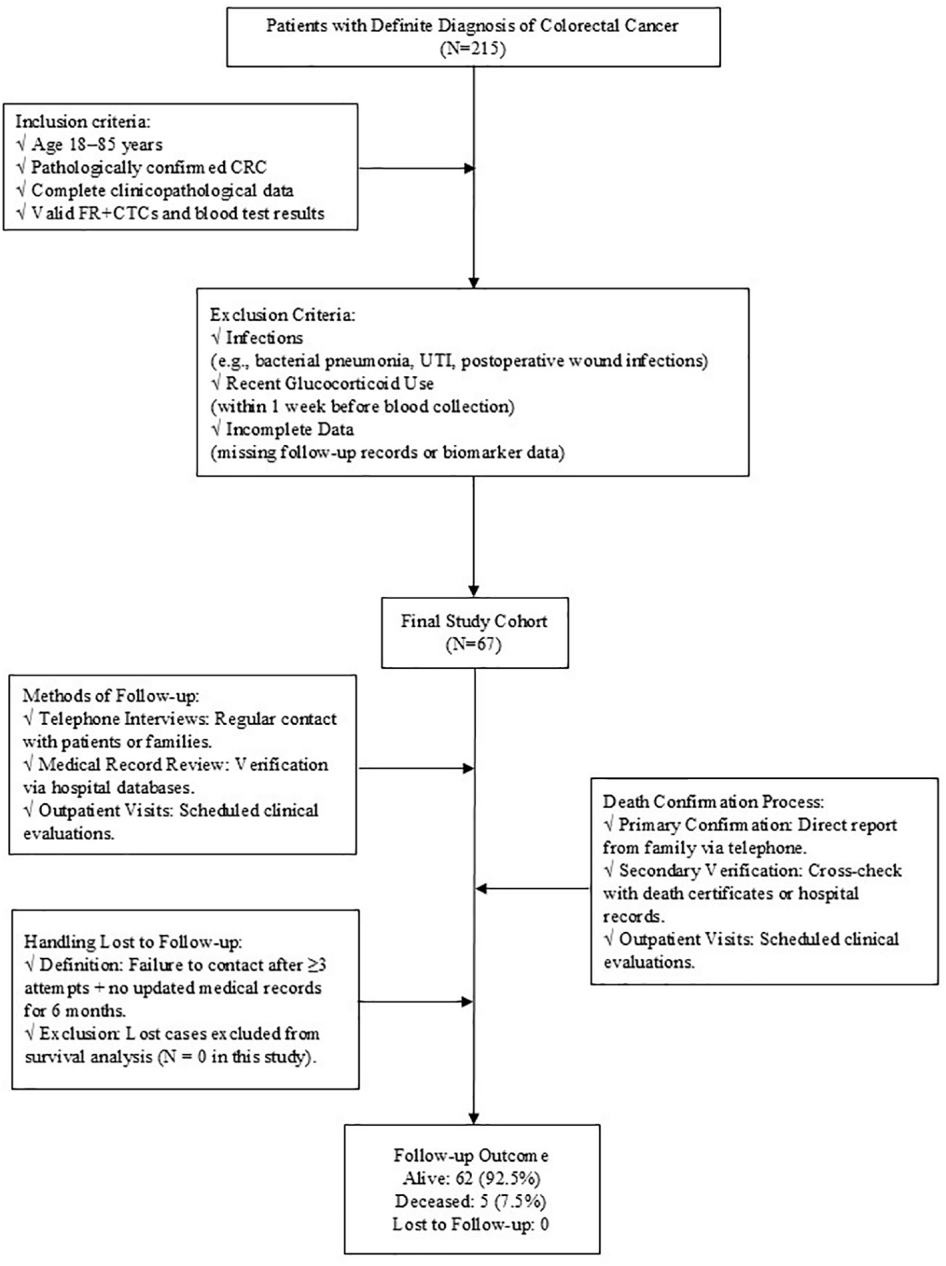
Patients Screening and Follow-up Workflow.

This is a retrospective study, which collected basic information and clinical data from patients who were admitted to The Central Hospital of Wuhan after radical surgery or radical chemoradiotherapy for CRC between January 2020 and December 2022. All patients were evaluated by clinical stage according to the American Joint Committee on Cancer (AJCC) 8th version TNM staging system (21). Inclusion criteria: (1) 18~85 years old; (2) The diagnosis of CRC was confirmed through pathological examination; (3) Complete clinicopathological data and follow-up data were available; (4) Accurate levels of FR+CTCs and blood routine data were obtained. Exclusion criteria: (1) Infection (Significant infections that occur during treatment include bacterial pneumonia, urinary tract infections, post-operative wound infections, etc. All cases of infection were confirmed by clinical diagnosis (e.g., microbial culture, imaging evidence). The potential effects of these infections on immune function and inflammatory markers. Excluding these patients ensures that the inflammatory markers analyzed in this study, reflect tumor-related immune responses and not infection-induced changes.) that significantly affected the results of routine blood tests during treatment; (2) Patients who have been treated with corticosteroids, such as prednisolone, dexamethasone, methylprednisolone and so on within 1 week prior to blood collection, as these medications can alter immune response and potentially confound the analysis of inflammatory markers (**Figure 1**).

### 2.2 FR+CTCs detection methodology

FR+CTCs assay was performed using an immunomagnetic negative selection system combined with ligand-targeted quantitative PCR, as described in the manufacturer’s protocol (GNOMON Biotechnology Co., Kit Version 1.6). Key performance parameters of the assay were rigorously validated: Sensitivity: 79.6% (95% CI: 76.8–82.3%) for lung cancer detection, calculated using spiked CRC cell lines in healthy donor blood across three independent experiments. Specificity: 88.2% (95% CI: 85.1–90.8%), determined by comparing FR+CTC levels between 350 benign pulmonary disease patients and 560 confirmed lung cancer cases in a multicenter trial. Limit of Detection: 5 FR+CTC cells per 3 mL blood, validated through serial dilutions of cultured HCT116 cells (recovery rate: 85–115%, CV < 15%). Potential technical variability was addressed through standardized protocols: (1)Leukocyte Residuals: Dual CD45/CD14 immunomagnetic depletion reduced leukocyte contamination to ≤0.1% (verified by flow cytometry).(2) Erythrocyte Lysis Efficiency: Optimal lysis conditions (12 mL lysis buffer per 3 mL whole blood, 15 min incubation at 4°C) ensured >99% erythrocyte removal. (3) Pre-analytical Stability: Samples processed within 24 hours (4–10°C storage) showed no significant FR+CTC degradation (ΔCt < 0.5 vs. immediate processing). These protocols align with ISO 15189 guidelines for molecular diagnostics, and inter-lot reproducibility was confirmed (CV = 8.3% across 10 kit batches).

### 2.3 Data collection and processing

The clinical data of patients who met the inclusion and exclusion criteria were primarily collected via our hospital’s electronic medical record system. The parameters included: gender, age, FR+CTCs values, blood counts (including monocyte, lymphocyte, neutrophil and platelet counts), tumor location, tumor differentiation, vascular invasion, nerve invasion, and TNM stage, etc.

### 2.4 Follow-up

The enrolled patients were followed up until June 2024 or until their death or loss of follow-up. The follow-up strategies encompassed, but were not confined to, telephone consultations, review of medical records, and outpatient visits. Progression-free Survival (PFS) is defined as the interval from the enrolment of patients after radical surgery or radical chemoradiotherapy to the occurrence of disease progression or recurrence, with measurements recorded in months. Overall survival (OS) is defined as the duration from patient enrolment following radical surgery or radical chemoradiotherapy to the occurrence of a fatal event or the final follow-up, also measured in months. Tumor progression was evaluated using the RECIST criteria version 1.1.

### 2.5 Statistical analysis

Statistical analysis was conducted using SPSS 25.0 software. The correlation between MLR, NLR, PLR and FR+CTCs, and clinicopathological characteristics was assessed using Fisher exact test or Pearson’s chi-square test, as appropriate. The reference values for FR+CTCs are ascertained by the test kit instruction multi-center verification results. These values serve as the benchmark for the assessment of FR+CTCs concentrations within the scope of this study. OS was used as the state variable, and optimal cut-off value of MLR, NLR and PLR were determined using the Youden index, which are calculated as Sensitivity+Specificity-1. Receiver operating curves (ROC) were constructed and analyzed in separate groups. Kaplan-Meier survival curves were plotted to evaluate the PFS and OS of patients and the differences between groups were compared using the Log-rank test. Variance inflation factor (VIF) was used to evaluate the collinearity among variables. VIF of all variables included in the COX model had to be <5.0, indicating no significant collinearity. The independent prognostic significance of each parameter on PFS and OS was explored through univariate and multivariate COX regression analyses. The difference was considered statistically significant at *P <*0.05.

### 2.6 ML model construction

Overseas research has indicated that the sample size for constructing a ML-based prediction model should be at least 10 times the number of independent variables. This study identified five influencing factors. Consequently, the sample size was calculated to be 10 times this number, resulting in a total of 50 cases. Finally, 67 patients were enrolled in this study, satisfying the sample size requirement of the prediction model (22).

The prediction models were developed using R Studio, incorporating variables that showed a significant influence with *P* < 0.05. In this study, three ML algorithms prevalent in the medical field- Random Forest, Support Vector Machine, and Logistic Regression-were applied to construct the predictive model using R Studio and additional computational tools. The selected influencing factors were integrated into the system, with data formatting and attribute definition completed accordingly. The dataset is randomly divided into a 70% training set and a 30% test set, the training set was used to train the model, and the test set was used to assess the generalization ability of the model at the end of training. The performance of the predictive model was measured by selecting accuracy, sensitivity, specificity, Youden index, positive predictive value (PPV), negative predictive value (NPV), precision, recall, F1 value, and area under the curve (AUC) of ROC. The calibration curve was used to evaluate the calibration of the model, thereby identifying the optimal algorithm, that is, the most accurate predictive model.

RF is an ensemble algorithm comprising multiple decision tree classifiers. It constructs numerous decision trees trained on randomly sampled subsets of data and features, then aggregates predictions through majority voting or averaging, effectively mitigating overfitting risk (23). Suitable for classification, regression, and dimensionality reduction tasks, Random Forest demonstrates robustness to noise and missing values, outperforming single decision trees in prediction accuracy. In medicine, it is widely applied to model complex nonlinear relationships, such as tumor recurrence risk assessment and imaging feature classification.

SVM is a generalized linear classifier employing hinge loss functions and regularization for binary classification. By leveraging kernel functions (e.g., radial basis function, RBF), SVM maps data into higher-dimensional spaces to address nonlinear problems (24, 25). Its stability in balancing model complexity and generalizability makes it particularly effective for small-sample datasets. In medical research, SVM is commonly used for high-precision tasks such as gene expression profiling and pathological image recognition.

LR is a generalized linear model estimating the probability of event occurrence based on independent variables, especially suited for binary classification (26, 27). Its strengths lie in simplicity, computational efficiency, and ease of clinical deployment. In medicine, logistic regression is extensively utilized to develop risk scoring systems, such as cardiovascular event prediction and postoperative complication assessment.

## 3 Results

### 3.1 Patient baseline characteristics

After screening, a cohort of 67 patients was enrolled, comprising 37 males and 30 females with an average age of 61.37 years ranging from 25 to 79 years. There were 32 patients with colon cancer and 35 patients with rectal cancer. According to TNM stage, there were 7 patients in stage I, 27 in stage II, and 33 in stage III. Among them, 46 patients had well-differentiated tumors, 14 patients had poorly-differentiated tumors, and 7 patients had unknown degree of differentiation. Of the patients, 41 had no vascular invasion, while 26 had vascular invasion. Similarly, 36 had no nerve invasion and 31 had nerve invasion. (**Table 1**) The average follow-up period was 30.28 months, with a median follow-up time of 30 months. Up to the follow-up deadline, 22 patients experienced disease progression, 62 survived, and 5 succumbed to the disease.

**Table 1:** Clinicopathologic variables in patients with colorectal cancer.

Variables	n	%
Gender			
Female	30	44.8
Male	37	55.2
Age			
<60	29	43.3
≥ 60	38	56.7
Location			
Colon	32	47.8
Rectum	35	52.2
Differentiation			
Well	46	68.7
Poor	14	20.9
Unknown	7	10.4
T stage			
T1	1	1.5
T2	7	10.4
T3	44	65.7
T4	15	22.4
N stage			
N0	34	50.8
N1	21	31.3
N2	12	17.9
Clinical stage			
I	7	10.4
II	27	40.3
III	33	49.3
Vascular invasion			
No	36	53.8
Yes	31	46.2
Nerve invasion			
No	41	61.2
Yes	26	38.8
MLR		
<0.234	35	52.2
≥0.234	32	47.8
NLR		
<1.822	29	43.3
≥1.822	38	56.7
PLR		
<206.358	51	76.1
≥206.358	16	23.9
FR+CTCs		
<8.7	46	68.7
≥8.7	21	31.3

### 3.2 Diagnostic Performance of MLR, NLR, PLR and FR+CTCs

**Figure 2:**
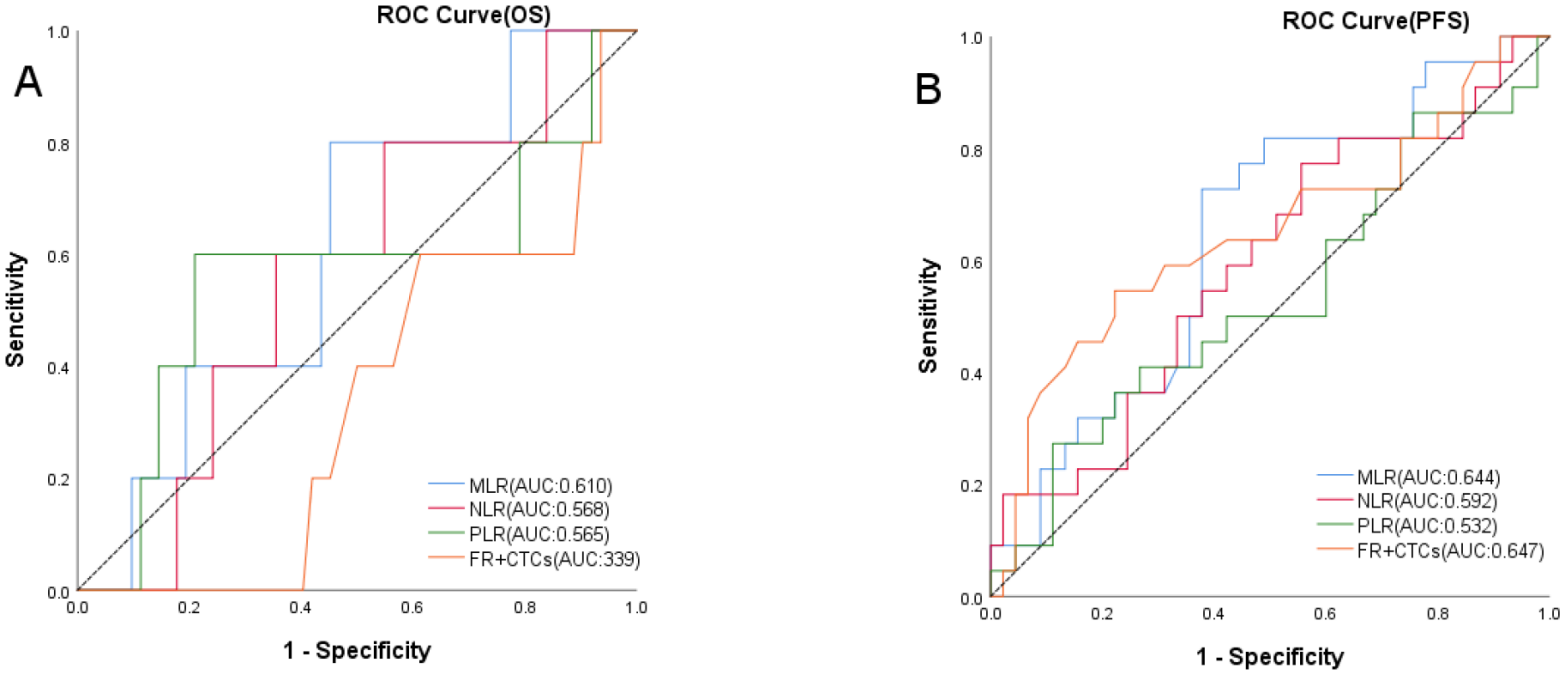
ROC curves of OS **(A)** in MLR, NLR, PLR and FR+CTCs groups, PFS **(B)** in MLR, NLR, PLR and FR+CTCs groups.

Using OS as the state variable, the area under the ROC curve (AUC) in the MLR group was 0.610 (95% CI: 0.385~0.834). The area under the ROC curve (AUC) in the NLR group was 0.568 (95% CI: 0.131~0.546). The area under the ROC curve (AUC) in the PLR group was 0.565 (95% CI: 0.340~0.796). The area under the ROC curve (AUC) in the FR+ CTCs group was 0.339 (95% CI: 0.131~0.546). (**Figure 2A**) Using PFS as the state variable, the area under the ROC curve (AUC) in the MLR group was 0.644 (95% CI: 0.505~0.782). The area under the ROC curve (AUC) in the NLR group was 0.592 (95% CI: 0.445~0.739). The area under the ROC curve (AUC) in the PLR group was 0.534 (95% CI: 0.381~0.688). The area under the ROC curve (AUC) in the FR+ CTCs group was 0.647 (95% CI: 0.497~0.798). (**Figure 2B**) Based on the Youden index of the ROC curve, the cut-off of MLR is 0.234, MLR <0.234 was classified as the low MLR group, and MLR ≥ 0.234 as the high MLR group. The cut-off of NLR is 1.822, NLR <1.822 was classified as the low NLR group, and NLR ≥ 1.822 as the high NLR group. The cut-off of PLR is 206.358, PLR <206.358 was classified as the low PLR group, and PLR ≥ 206.358 as the high PLR group. According to the instructions for the FR+CTCs assay kit used at our institution, FR+CTCs ≥8.7 FU/3mL was selected as the reference value. Therefore, in this study, FR+CTCs <8.7FU/3mL were classified as the low FR+CTCs group, and FR+ CTCs ≥8.7FU/3mL as the high FR+CTCs group. 35 patients had an MLR <0.234 and 32 patients had an MLR ≥0.234. 29 patients had an NLR <1.822 and 38 patients had an NLR ≥1.822. 51 patients had an PLR <206.358 and 16 patients had an PLR ≥206.358. 46 patients had FR+CTCs <8.7 FU/3mL and 21 patients had FR+CTCs ≥8.7 FU/3mL (**Table 1**).

### 3.3 The correlation between MLR, NLR, PLR, FR+CTCs and clinicopathological characteristics

Based on the established cut-off values, the study population was divided into two groups: a low MLR group consisting of 35 patients and a high MLR group with 32 patients. In the NLR group, there were 29 patients in the low NLR group and 38 patients in the high NLR group. In the PLR group, there were 51 patients in the low PLR group and 16 patients in the high PLR group. Similarly, there were 46 patients in the low FR+ CTCs group and 21 patients in the high FR+ CTCs group. In the MLR group, there was a statistical difference in clinical stage, with a statistical significance of P=0.028. However, no significant differences were observed in gender, age, tumor location, tumor differentiation, tumor invasion depth, lymph node involvement, vascular invasion, or nerve invasion between the groups (*P* > 0.05). No statistical differences were observed between the NLR and PLR groups and clinicopathological features (P > 0.05). In the FR+CTCs group, the age of patients was significantly different (*P*=0.009). There was no statistical difference in other characteristics of the patients (*P*>0.05) (**Table 2**).

**Table 2:** Comparison of different clinicopathological features with MLR and FR+CTCs.

Variables	MLR		*P*	NLR		*P*	PLR		*P*	FR+ CTCs (FU/3mL)		*P*
	<0.234 n=35(%)	≥0.234 n=32(%)		<1.822 n=29(%)	≥1.822 n=38(%)		<206.358 n=51 (%)	≥206.358 n=16 (%)		<8.7 n=46(%)	≥8.7 n=21(%)	
Gender			0.514			0.994			0.925			0.398
Female	17(48.6)	13(40.6)		13(44.8)	17(44.7)		23(45.1)	7(43.8)		19(41.3)	11(52.4)	
Male	18(51.4)	19(59.4)		16(55.2)	21(55.3)		28(54.9)	9(56.2)		27(58.7)	10(47.6)	
Age			0.361			0.471			0.593			0.009
<60	17(48.6)	12(37.5)		14(48.3)	15(39.5)		23(45.1)	6(37.5)		15(32.6)	14(66.7)	
≥60	18(51.4)	20(62.5)		15(51.7)	23(60.5)		28(54.9)	10(62.5)		31(67.4)	7(33.3)	
Location			0.530			0.570			0.130			0.117
Colon	18(51.4)	14(43.8)		15(51.7)	17(44.7)		27(52.9)	5(31.3)		19(41.3)	13(61.9)	
Rectum	17(48.6)	18(56.3)		14(48.3)	21 (55.3)		24(47.1)	11(68.7)		27(58.7)	8(38.1)	
Differentiation			0.323			0.343			0.909			0.775
Well	21(60)	25(78.1)		18(62.1)	28(73.7)		34(66.7)	12(75)		30(65.2)	16(76.2)	
Poor	9(25.7)	5(15.6)		6(20.7)	8(21.1)		11(21.6)	3(18.8)		11(23.9)	3(14.3)	
Unknown	5(14.3)	2(6.3)		5(17.2)	2(5.2)		6(11.7)	1(6.2)		5(10.9)	2(9.5)	
T stage			0.466			0.740			0.700			0.580
T1	1(2.9)	0(0)		0(0)	1(2.6)		1(2.0)	0(0)		0(0)	1(4.8)	
T2	2(5.7)	5(15.6)		3(10.3)	4(10.5)		5(9.8)	2(12.5)		5(10.9)	2(9.5)	
T3	23(65.7)	21(65.6)		21(72.4)	23(60.5)		32(62.7)	12(75)		30(65.2)	14(66.7)	
T4	9(25.7)	6(18.8)		5(17.3)	10(26.4)		13(25.5)	2(12.5)		11(23.9)	4(19)	
N stage			0.068			0.482			0.084			0.301
N0	13(37.1)	21(65.6)		13(44.8)	21(55.3)		24(47.1)	10(62.5)		22(47.8)	12(57.1)	
N1	14(40)	7(21.9)		9 (31.0)	12(31.6)		15(29.4)	6(37.5)		17(37)	4(19)	
N2	8(22.9)	4(12.5)		7(24.2)	5(13.1)		12(23.5)	0(0)		7(15.2)	5(23.8)	
Clinical stage			0.028			0.599			0.388			0.328
I	1(2.9)	6(18.8)		2(6.9)	5(13.1)		4(7.9)	3(18.7)		6(13)	1(4.8)	
II	12(34.3)	15(46.9)		11(37.9)	16(42.2)		20(39.2)	7(43.8)		16(34.8)	11(52.4)	
III	22(62.8)	11(34.4)		16(55.2)	17(44.7)		27(52.9)	6(37.5)		24(52.2)	9(42.9)	
Vascular invasion			0.924			0.483			0.732			0.498
No	19(54.3)	17(53.1)		17(58.6)	19(50)		28(54.9)	8(50)		26(56.5)	10(47.6)	
Yes	16(45.7)	15(46.9)		12(41.4)	19(40)		23(45.1)	8(50)		20(43.5)	11(52.4)	
Nerve invasion			0.477			0.898			0.477			0.646
No	20(57.1)	21(65.6)		18(62.1)	23(60.5)		30(58.8)	11(68.7)		29(63)	12(57.1)	
Yes	15(42.9)	11(34.4)		11(37.9)	15(39.5)		21(41.2)	5(31.3)		17(37)	9(42.9)	

### 3.4 Comparison of prognosis different MLR, NLR, PLR and FR+CTCs groups

**Figure 3:**
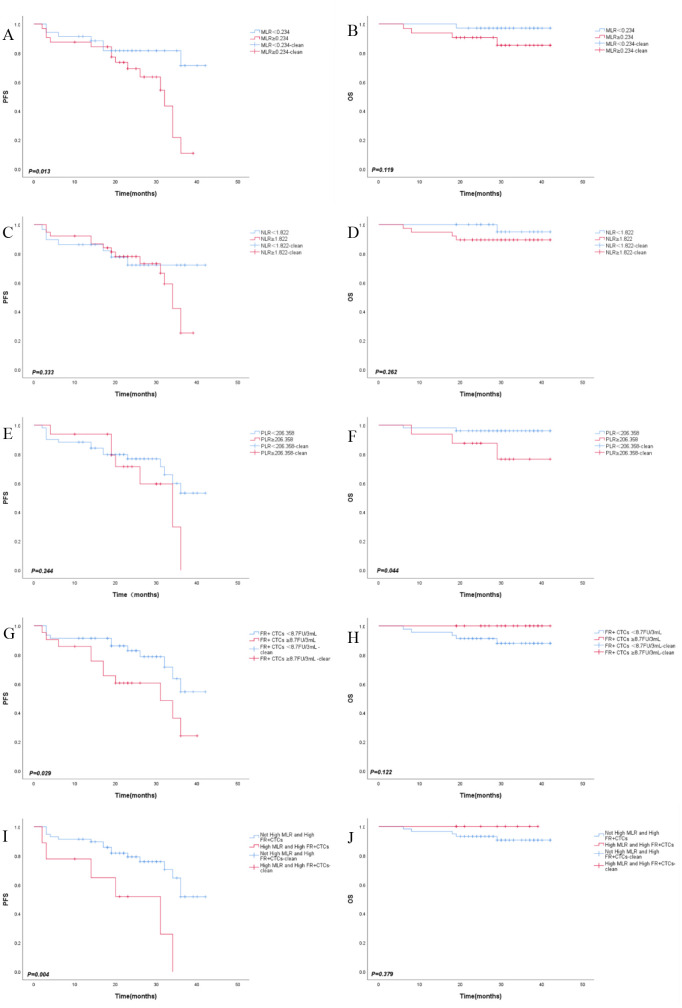
Kaplan-Meier survival curves of PFS **(A)**, OS **(B)** in MLR group, PFS **(C)**, OS **(D)** in NLR group, PFS **(E)**, OS **(F)** in PLR group, PFS **(G)**, OS **(H)** in FR+CTCs group, PFS **(I)**, OS **(J)** in combined MLR amd FR+CTCs group.

In our study, we investigated the predictive value of MLR, NLR, PLR and FR+CTCs on clinical prognosis in CRC. Separate Kaplan-Meier survival analyses were conducted for the MLR and FR+CTCs group. The results indicated that PFS was longer in the low MLR group compared to the high MLR group (35.58 ± 2.00 months vs. 27.24 ± 2.10 months, *P*=0.013, **Figure 3A**). The OS was also longer in the low MLR group than in the high MLR group (41.34 ± 0.65 months vs. 38.40 ± 1.73 months, *P*=0.119, **Figure 3B**). Regarding NLR, although the low NLR group showed a trend toward improved PFS (33.57 ± 2.78 months vs. 30.03 ± 1.84 months, *P* = 0.333, **Figure 3C**) and OS (41.35 ± 0.63 months vs. 38.92 ± 1.49 months, *P* = 0.262, **Figure 3D**), these differences were not statistically significant. However, no significant difference in PFS was observed between low and high PLR groups (32.61 ± 2.03 months vs. 28.49 ± 2.69 months, P = 0.244, **Figure 3E**). In contrast, PLR demonstrated a significant association with OS, with the low PLR group exhibiting markedly longer OS compared to the high PLR group (40.83 ± 0.82 months vs. 36.95 ± 2.66 months, P = 0.044, **Figure 3F**). However, there was no significant difference. The PFS was longer in the low FR+CTCs group than in the high FR+CTCs group (34.24 ± 1.98 months vs. 26.56 ± 2.94 months, *P*=0.029, **Figure 3G**); There was no significant difference in OS between the low and high FR+CTCs groups (*P*=0.122, **Figure 3H**).

### 3.5 Combined with MLR and FR+CTCs to evaluate the prognosis

These results suggest that NLR and PLR have no significant prognostic effect on CRC. Ultimately, our study aimed to determine the prognostic threshold when combining MLR and FR+CTCs in CRC patients. MLR and FR+ CTCs were divided into high group and low group and we reintegrated MLR and FR+CTCs into ①Not High MLR and High FR+CTCs group (n=58) and ②High MLR and High FR+CTCs group (n=9). We conducted survival analyses for both PFS and OS for each group and plotted the corresponding survival curves. The average PFS for the “Not High MLR and High FR+CTCs” group was significantly longer at 33.53 ± 1.77 months compared to the “High MLR and High FR+CTCs” group at 21.82 ± 4.63 months (*P*=0.004, **Figure 3I**). However, there was no significant difference in OS between the two groups (*P*=0.379, **Figure 3J**).

### 3.6 Cox regression analyses

All variables included in the COX model were evaluated by VIF, with VIF <4.0 (threshold VIF=5.0), indicating no significant collinearity (**Table 3**). Furthermore, A univariate analysis was performed on various clinicopathologic features, revealing that T stage (HR, 95%CI: 2.865, 1.287~6.377, *P*=0.010), nerve invasion (HR, 95%CI: 1.309, 0.565-3.035, *P*=0.024), MLR (HR, 95%CI: 2.987, 1.194-7.474, *P*=0.019), and FR+CTCs (HR, 95%CI: 2.429, 1.052-5.609, *P*=0.038) significantly influenced PFS (**Table 4**). Notably, only T stage was a significant predictor of OS (HR, 95%CI: 12.814, 1.482-110.802, *P*=0.02, **Table 4**). Consequently, we integrated T stage, nerve invasion, MLR, and FR+CTCs into a multivariate COX analysis. This analysis demonstrated that T stage (HR, 95%CI: 3.338, 1.298-8.585, *P*=0.012), MLR (HR, 95%CI: 5.152, 1.806-14.696, *P*=0.002), and FR+CTCs (HR, 95%CI: 3.440, 1.355-8.732, *P*=0.009) were superior predictors of PFS (**Table 5**), with T stage showing better performance in assessing OS (HR, 95%CI: 9.756, 1.076-88.470, *P*=0.043, **Table 5**).

**Table 3:** Variance inflation factor analysis result.

	Unnormalized coefficient		Standardization coefficient			Collinearity statistics	
	B	Standard error	Beta	t	Significance	Tolerance	VIF
Constant	40.445	7.519		5.379	0.000		
Gender	1.466	1.983	0.094	0.740	0.463	0.925	1.081
Age	-2.497	2.146	-0.159	-1.164	0.250	0.796	1.257
Location	-2.616	2.141	-0.168	-1.222	0.227	0.786	1.272
Differentiation	-1.075	1.479	-0.093	-0.727	0.471	0.909	1.100
T stage	-2.499	1.917	-0.198	-1.304	0.198	0.644	1.553
N stage	-2.048	2.414	-0.200	-0.849	0.400	0.267	3.751
Clinical stage	2.031	2.810	0.174	0.722	0.473	0.255	3.922
Vascular invasion	1.444	2.186	0.092	0.660	0.512	0.757	1.322
Nerve invasion	-2.560	2.162	-0.160	-1.184	0.242	0.810	1.234
MLR	-3.115	2.415	-0.200	-1.290	0.203	0.618	1.618
NLR	0.950	2.310	0.060	0.411	0.683	0.686	1.457
PLR	-3.319	2.703	-0.182	-1.228	0.225	0.677	1.477
CTC	-0.555	2.303	-0.033	-0.241	0.810	0.788	1.269

a. Dependent variable: OS.

**Table 4:** Univariate survival analysis of PFS and OS.

Variables	PFS			OS		
	HR	95%CI	*P*	HR	95%CI	*P*
Gender			0.483			0.869
Female	1.363	0.537~3.239		1.163	0.194~6.976	
Male						
Age			0.927			0.273
<60	0.962	0.415~2.229		3.411	0.380~30.623	
≥ 60						
Location			0.093			0.624
Colon	0.462	0.188~1.138		0.639	0.107~3.828	
Rectum						
Differentiation			0.516			0.880
Well	1.238	0.650~2.359		1.107	0.296~4.138	
Poor						
Unknown						
T stage			0.010			0.020
T1	2.865	1.287~6.377		12.814	1.482~110.802	
T2						
T3						
T4						
N stage			0.472			0.835
N0	1.230	0.700~2.160		0.880	0.265~2.921	
N1						
N2						
Clinical stage			0.430			0.490
I	1.333	0.654~2.717		1.707	0.374~7.784	
II						
III						
Vascular invasion			0.530			0.741
No	1.309	0.565~3.035		0.740	0.124~4.428	
Yes						
Nerve invasion			0.024			0.337
No	2.730	1.145~6.511		2.401	0.401~14.374	
Yes						
MLR			0.019			0.159
<0.234	2.987	1.194~7.474		4.842	0.540~43.409	
≥0.234						
NLR			0.344			0.289
<1.822	1.543	0.628~3.793		3.270	0.365~29.272	
≥1.822						
PLR			0.256			0.071
<206.358	1.693	0.683~4.197		5.213	0.869~31.281	
≥206.358						
FR+CTCs			0.038			0.366
<8.7	2.429	1.052~5.609		0.027	0~69.160	
≥8.7			0.483			0.869

**Table 5:** Multivariate COX analysis of PFS and OS.

Variables	PFS			OS		
	HR	95%CI	*P*	HR	95%CI	*P*
T stage	3.338	1.298~8.585	0.012	9.756	1.076~88.470	0.043
Nerve invasion	1.498	0.579~3.873	0.405	1.601	0.241~10.650	0.626
MLR	5.152	1.806~14.696	0.002	4.812	0.517~10.650	0.167
FR+CTCs	3.440	1.355~8.732	0.009	0	0~	0.976

### 3.7 ML model construction and performance evaluation

**Figure 4:**
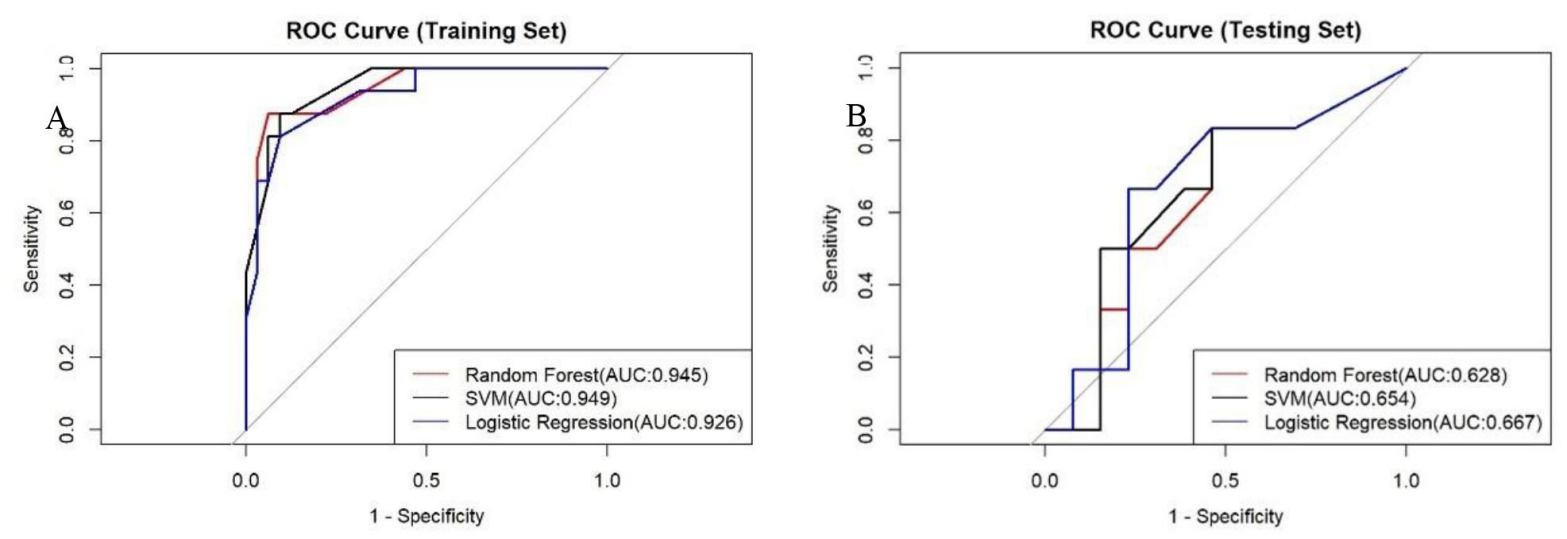
ROC curves for the three models on the training set **(A)** and testing set **(B)**.

**Figure 5:**
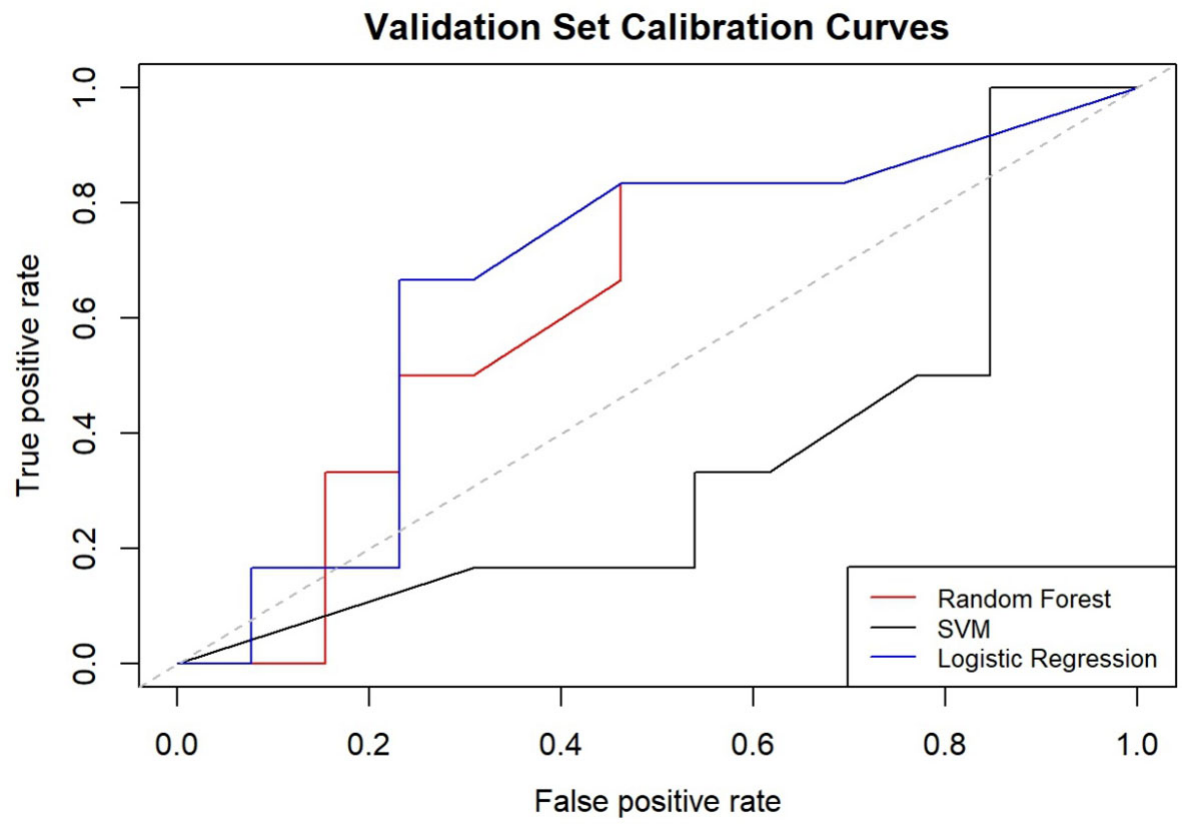
Calibration curves for the three models.

In the development of our predictive model for PFS, we identified significant risk factors, including MLR, FR+CTCs, T stage, and nerve invasion. We employed a ML methodology for model construction and utilized three distinct algorithms: RF, SVM, and LR. The dataset was randomly partitioned into a 70% training set and a 30% testing set. Each of the three models was then used to extract key performance metrics for comparative analysis. The performance metrics of each model are detailed in **Table 6**, their ROC curves are presented in **Figure 4**, and the calibration curves of each model are shown in **Figure 5**.

**Table 6:** Comprehensive assessment of the predictive power of models.

Models	Accuracy	Sensitivity	Specificity	Youden Index	PPV	NPV	Precision	Recall	F1 score	AUC
RF	0.63	0.69	0.5	0.19	0.75	0.43	0.43	0.5	0.46	0.63
SVM	0.32	0.5	0.23	-0.27	0.23	0.5	0.23	0.5	0.32	0.65
LR	0.63	0.4	0.71	0.11	0.33	0.77	0.4	0.33	0.36	0.67

RF model excelled in accuracy (0.63), sensitivity (0.69), and PPV (0.75), making it an ideal choice for scenarios where high sensitivity and reliability are paramount. In contrast, the LR model, on the other hand, demonstrated a robust overall diagnostic capability achieving the highest AUC of 0.67, particularly excelling in specificity (0.71) and NPV (0.77), thus, being well-suited for scenarios aiming to minimize false positives. The SVM model demonstrated a balanced performance across all metrics. Regarding precision and recall, the RF model stood out with the highest precision (0.43) and recall (0.5). The F1 scores of the three models ranged from 0.32 to 0.46, with a closer F1 value to 1 indicating superior model prediction model prediction. The RF model’s F1 score was 0.46, the highest among the three models, signifying its best prediction performance. In the training dataset, the AUC values of the three models were relatively close, with the SVM model having a peak at 0.949. However, in the test dataset, the LR model outperformed with the highest AUC value of 0.67, suggesting it had superior predictive accuracy over the other models.

When calibrating the three models, the curve of RF model was closer to the diagonal line, indicating relatively better calibration performance. Conversely, the SVM and LR models’ curves were further away from the diagonal line, indicating less effective calibration. The comprehensive analysis concludes that the RF model outperforms the other three models in terms of overall performance and delivers the most accurate predictions.

## 4 Discussion

In recent years, there has been a surge in research focused on tumor microenvironments, highlighting the intricate interactions between the components within these environments t and tumor cells. The tumor microenvironment is now recognized as a critical factor in tumor formation, progression, recurrence, and metastasis (2). The impact of inflammatory cells on tumors has garnered significant interest, yet the precise physiological mechanisms behind this influence remain to be fully elucidated. In the process of tumor development, a prevailing hypothesis in the field posits that errors in gene repair mechanisms can trigger the abnormal activation of proto-oncogenes and the silencing of tumor suppressor genes. This, in turn, may enhance the transcriptional activities of inflammatory mediators, leading to a substantial escalation in inflammatory responses within the tumor microenvironment. Consequently, this heightened inflammation can profoundly affect the state of the tumor microenvironment, with implications for cancer development and behavior (28).

MLR serves as a gauge for the body’s immune response to malignancies. Jakubowska et al. demonstrated that postoperative MLR as independent prognostic markers for 5-year disease-free survival in colorectal cancer patients (29). Huang et al. found that colorectal cancer patients with preoperative MLR positivity (>0.24) exhibited poorer prognosis, and the combination of MLR with other tumor markers significantly improved prognostic evaluation, identifying it as an independent risk factor for colorectal cancer (30). CHAN et al. discovered that an elevated LMR was associated with better OS and could be utilized as an independent predictor of OS in CRC patients undergoing radical surgery (31). Shen et al. identified MLR as a risk factor for distant metastasis in CRC patients; therefore, MLR can serve as an effective prognostic marker for metastatic CRC cases (32). A Meta-analysis encompassing 15 studies revealed that a high LMR was associated with superior survival rates and was a significant predictor for assessing prognosis (11). Consistent with that in our study, patients with elevated MLR indicated a poorer prognosis and significantly shorter PFS compared to those with low MLR, and low MLR often being associated with a more favorable prognosis. Encouragingly, both univariate and multivariate analyses demonstrated that MLR is an independent predictor of PFS.

In this study, although the neutrophil-to-lymphocyte ratio (NLR) and platelet-to-lymphocyte ratio (PLR) have been demonstrated to possess prognostic value in several malignancies—including non-small cell lung cancer (5), breast cancer (6), hepatocellular carcinoma (7), and gastric cancer (8)—our analysis did not reveal a significant prognostic impact of these markers in colorectal cancer (CRC). This discrepancy may be attributable to several factors. First, the relatively small sample size in our study may have reduced the statistical power necessary to detect significant associations between NLR, PLR, and patient outcomes. Second, the retrospective design may have introduced potential confounding factors and selection bias (e.g., variations in individual patient characteristics and treatment regimens) that could obscure the true prognostic utility of these inflammatory markers. Third, the specific clinical characteristics of our study population—such as the distribution of tumor stages and other clinicopathological features—might have limited the applicability of NLR and PLR as robust prognostic indicators in this setting. Notably, while previous studies have underscored the prognostic value of various inflammatory indices in CRC, our findings indicate that MLR, especially when combined with FR+CTCs, provides a more reliable predictive performance. Future large-scale, prospective studies are warranted to further elucidate the roles of NLR and PLR in CRC prognosis.

Monocytes differentiate into tumor-associated macrophages (TAM), an important component of anti-tumor immunity. Macrophages are broadly classified according to their function into two major groups: M1 (classical) and M2 (alternative). M1 macrophages that participate in the inflammatory response, are responsible for the efficient clearance of pathogens and are actively involved in the initiation and maintenance of anti-tumor immunity. In contrast, M2 macrophages have important roles in regulating anti-inflammatory responses, promoting wound healing, and expressing pro-tumor properties (33).M2 macrophages, stimulated by IL-4 and IL-13, secrete IL-10, TGF-β, and chemokines, which are involved in the remodeling of the tumor stroma and promote angiogenesis and tumor cell infiltration, thereby accelerating tumor progression (34, 35). In tumor tissues, M2 macrophages are the main expression cells of TAM, and their high aggregation is closely related to the poor prognosis of patients. At the same time, lymphocytes play a key role in anti-tumor immune responses and tumor immune surveillance. Lymphocytes can reflect the immune status of the body to a certain extent, and if lymphocytes are reduced, it suggests that the body’s immune defense ability is reduced, which may lead to a reduction in the inhibitory efficacy of the proliferation and differentiation of tumor cells.

Traditional postoperative surveillance is based on imaging and tumor marker assays, and often falls short in the timely and precise detection of residual cancer cells. In contrast, CTCs have the potential to anticipate disease recurrence prior to conventional clinical or radiographic indicators, thereby facilitating the opportunity for preemptive therapeutic intervention. The principal mechanism through which CTCs facilitate distant metastasis remains the subject of ongoing research. Some researchers believe that CTCs undergo Epithelial-Mesenchymal Transition (EMT), enabling tumor cells to degrade extracellular matrix components and infiltrate the vascular wall to gain access to the circulatory system. However, it is a subset of CTCs in circulation that do not undergo EMT (36). A retrospective analysis of 36 cases found that in patients with primary CRC, the presence of CTCs in the peripheral blood is indicative of a poor prognosis (37). Additionally, it has been observed that non-metastatic colon cancer patients with positive CTCs expression have significantly shorter PFS than CTCs-negative patients, suggesting that CTCs serve as a robust predictor for prognostic assessment (38). In China, some researchers have found that stage II CRC patients with postoperative positive CTCs exhibit shorter PFS compared to CTCs-negative patients, indicating that postoperative CTCs can be used to assess the prognosis of stage II CRC patients (39). The folate receptor (FR) family contains multiple subtypes, with FRα demonstrating elevated expression in a variety of solid tumors (40, 41). In CRC, FRα expression is significantly increased, and higher FRα levels are associated with more aggressive tumor behavior and poorer patient prognosis (42). Therefore, FR can be employed as a highly sensitive biomarker for the detection of CTCs in the peripheral blood of oncology patients. This study, by quantitative analysis of FR+CTCs, suggests that patients with elevated FR+CTCs levels have shorter PFS and a worse prognosis.

Currently, the combination of inflammatory biomarkers and CTCs has emerged as a novel approach to evaluate the prognosis of tumor patients. DECIORGI et al. identified a significant correlation between the MLR and CTCs levels, utilizing this combination as a prognostic tool in primary breast cancer (9). Qian et al. employed the NLR in conjunction with CTCs to predict CRC prognosis, revealing that patients exhibiting elevated NLR and CTCs values had a significantly worse prognosis (43). HU et al. suggested that the Systemic Immune-Inflammation Index (SII) may offer a more accurate reflection of the interplay between inflammationory and immune response dynamics compared to other indices such as PLR and NLR, with high SII patients exhibiting increased relapse rates and reduced survival durations (44). Despite these findings, the combined predictive value of MLR and CTCs in assessing the prognosis of CRC patients post-radical surgery or radical chemoradiotherapy has not been conclusively established. Given the established link between MLR and tumor progression, along with the prognostic predictive capabilities of CTCs, this study aims to combine MLR with CTCs to predict survival outcomes in CRC patients.

The Chen team found that among four ML models, LR, RF, Classification and Regression Decision Tree (CART), and SVM, the LR model was the best predictor of tumor recurrence in colorectal cancer patients post Stage II and Stage III surgery. The authors also enhanced the predictive accuracy by combining the LR model with nomograms (45). Internationally, Roshanaei G et al. found in a retrospective study that the RF model predicts the prognosis of colorectal cancer patients more accurately than traditional COX analysis (46). Achilonu O J et al. used six ML models to analyze the prognosis of CRC patients in South Africa, with Artificial Neural Networks (ANNs) showing the highest AUC values and the best predictive performance in predicting recurrence and survival rates (47). In patients with early-onset non-metastatic colorectal cancer, Zhao Hongmei et al. employed a nomogram model for risk- stratification, offering a more personalized approach compared to the traditional TNM stage (48). The optimal ML model for colorectal cancer prognosis varies among scholars worldwide, influenced by the different factors considered by each researcher. Some investigate factors such as psychiatric, psychological, and environmental factors, while others focus on specific immunohistochemical markers in pathology or incorporate clinicopathological characteristics (45–49). In our study, we used factors with independent predictive significance for PFS, as identified by COX analysis, as the basis for our prediction model. We constructed three ML models, compared their key performance indicators, and compared the advantages and disadvantages of each model. In this study, the random forest (RF) model achieved a modest overall performance, with an accuracy of 0.63 and a sensitivity of 0.69. These performance metrics are in line with previous prognostic models in colorectal cancer (CRC), as exemplified by Chen et al., who reported AUC values ranging from 0.581 to 0.678 across four machine learning approaches (46). Notably, the RF model emphasizes a high specificity (0.71) and a positive predictive value (PPV of 0.75), which are pivotal in minimizing false-positive outcomes. In a clinical setting, high specificity and PPV are particularly valuable because they ensure that patients identified as high-risk are more likely to require aggressive intervention, thereby reducing the risk of overtreatment. This is crucial given that overtreatment may lead to unnecessary exposure to the adverse effects of therapy and incur significant economic burdens. In contrast, if the clinical objective were to reliably identify negative cases, prioritizing specificity and negative predictive value (NPV) over sensitivity and PPV, an alternative model such as logistic regression (LR) might be preferable. Similarly, while the support vector machine (SVM) model demonstrated balanced performance across various indicators, it did not significantly outperform the other models. Therefore, considering the study’s focus on early recurrence and metastasis prediction, the RF model appears to be the optimal choice for stratifying patients and guiding clinical decisions. The adoption of such a model in clinical workflows could facilitate more tailored treatment strategies. By accurately identifying low-risk patients who may safely avoid aggressive adjuvant therapies, clinicians can optimize resource allocation and improve patient management, particularly in resource-constrained settings.

In clinical practice, the TNM stage, as a cornerstone in oncology classic assessment system, effectively delineates the disease’s progression and serves as a pivotal instrument in determining the necessity for adjuvant therapy and in prognostic assessment. However, in the precision medicine era, reliance on the traditional TNM stage system alone is inadequate for the nuanced demands of individualized treatment planning for oncology patients. While the TNM guidelines suggest a direct relationship between stage advancement and prognosis deterioration, evidence indicates that stage II patients may have a poorer prognosis than stage IIIa patients (50), suggesting that the system may not effectively guide the precision and individualization of subsequent treatment strategies. Notably, the TNM system’s broad categorizations are particularly limiting when determining the need for adjuvant therapy and the specific adjuvant treatment strategies. The TNM system solely considers tumors, lymph nodes, and metastases, yet previous considers have demonstrated that certain clinicopathological characteristics, such as tumor histological type and perineural invasion, can also exert a significant impact on the prognosis of colorectal cancer patients (51). This indicates that TNM system is overly generalized. By employing ML, we can integrate a multitude of influencing factors and consider them in a comprehensive manner, facilitating the implementation of more individualized and targeted therapeutic interventions.

## 5 Limitations

The findings of this study are based on a single-center, retrospective cohort analysis of patients who undergo definitive surgical resection or radical oncologic therapy. While this study design offers valuable “real-world” clinical practice, it lacks the methodological rigor of randomized controlled trials (RCTs), which are characterized by strict patient enrollment criteria, standardized treatment protocols, and meticulous follow-up procedures. Furthermore, due to the limited sample sizes and short follow-up duration in this study, our conclusions require validation in studies with larger sample size and extended follow-up periods before they can be applied to predict patient survival in clinical settings. Additionally, for patients with colorectal cancer post-radical surgery or chemoradiotherapy, specific testing time points for FR+CTCs and blood counts were not standardized, with some tests being conducted at considerable distances from the time of radical treatment. Only the initial postoperative test data were obtained in this study, yet blood cell counts and FR+CTCs are variables that fluctuate over time. The correlation between these dynamic changes and CRC prognosis remains unclear, with the underlying pathophysiological mechanisms largely unexplored. Future research should aim to clarify these dynamics and their implications for CRC prognosis. Finally, the correlation analysis of MLR, NLR and PLR was carried out in this study, but the results showed that NLR and PLR had no significant significance in the prognosis evaluation of CRC.A comprehensive evaluation of these parameters in conjunction with MLR would provide a more detailed understanding of their individual predictive capabilities and limitations, thereby enhancing the precision and accuracy of prognostic evaluations. The current model relies solely on FR+CTCs and MLR as predictive features. While both markers have demonstrated prognostic value, their combined predictive power may not fully capture the multifactorial nature of CRC progression. One key limitation is the reliance on these static measurements; incorporating dynamic measurement data could improve performance. For example, tracking changes in FR+CTCs levels over time or integrating dynamic monitoring data of other clinical variables may allow the model to better capture disease progression and the true prognostic state of the patient (47). This study is based on a single-center, retrospective cohort with a limited sample size. Prospective, multi-center studies with larger cohorts and extended follow-up periods are essential to validate the model’s reliability and ensure its generalizability across diverse patient populations. The integration of machine learning models into routine clinical practice remains challenging due to issues of interpretability and data integration. Future research should focus on developing explainable AI techniques to clarify the decision-making processes within the model. Furthermore, the establishment of robust electronic health record systems and enhanced interdisciplinary collaboration between clinicians and data scientists will be crucial for successful clinical translation. This study is constrained by its small sample size and limited inclusion of influencing factors, with only a randomized split into training and test sets and no external validation. To address these limitations, it is necessary to expand the study population and incorporate a broader range of factors that impact the prognosis of patients with colorectal cancer, ideally, future studies should also aim to conduct external validation across multiple hospitals to strengthen the generalizability of the findings.

## 6 Conclusion

This study has demonstrated that the combined assessment of the MLR and FR+CTCs may be useful in predicting PFS in patients with CRC. However, the findings should be interpreted with caution due to the limited sample size, and further research is warranted to confirm their prognostic value in predicting OS. Among the predictive models developed, the RF model exhibited the most favorable performance in predicting post-treatment outcomes, particularly in terms of specificity and PPV. These characteristics suggest that the RF model has clinical utility in identifying patients who may be at lower risk and could potentially avoid unnecessary aggressive treatments, thereby reducing the risk of overtreatment. Moving forward, the integration of artificial intelligence (AI) into predictive modeling represents a promising avenue for enhancing prognostic accuracy. While challenges such as model interpretability, validation in larger cohorts, and clinical integration remain, the potential of AI-based models to improve individualized patient care and decision-making in oncology is significant and warrants further exploration.

## Data Availability

The original contributions presented in the study are included in the article/Supplementary Material. Further inquiries can be directed to the corresponding authors.
